# On the Unification of Common Actigraphic Data Scoring Algorithms

**DOI:** 10.3390/s21186313

**Published:** 2021-09-21

**Authors:** Piotr Biegański, Anna Stróż, Marian Dovgialo, Anna Duszyk-Bogorodzka, Piotr Durka

**Affiliations:** 1 Faculty of Physics, University of Warsaw, ul. Ludwika Pasteura 5, 02-093 Warsaw, Poland; a.stroz@uw.edu.pl (A.S.); marian.dovgialo@fuw.edu.pl (M.D.); duszykanna@gmail.com (A.D.-B.); p.durka@uw.edu.pl (P.D.); 2 Inter-Faculty Individual Studies in Mathematics and Natural Sciences, University of Warsaw, ul. Stefana Banacha 2C, 02-097 Warsaw, Poland

**Keywords:** actigraphy, sleep-wake scoring, Cole-Kripke algorithm, Sadeh algorithm, Sazonov algorithm, Webster algorithm, Scripps Clinic algorithm, UCSD algorithm

## Abstract

Actigraphy is a well-known, inexpensive method to investigate human movement patterns. Sleep and circadian rhythm studies are among the most popular applications of actigraphy. In this study, we investigate seven common sleep-wake scoring algorithms designed for actigraphic data, namely Cole-Kripke algorithm, two versions of Sadeh algorithm, Sazonov algorithm, Webster algorithm, UCSD algorithm and Scripps Clinic algorithm. We propose a unified mathematical framework describing five of them. One of the observed novelties is that five of these algorithms are in fact equivalent to low-pass FIR filters with very similar characteristics. We also provide explanations about the role of some factors defining these algorithms, as none were given by their Authors who followed empirical procedures. Proposed framework provides a robust mathematical description of discussed algorithms, which for the first time allows one to fully understand their operation and basics.

## 1. Introduction

Actigraphy is a well-recognized method for investigation of human activity, with broad applications spanning consumer technology [1], supplementary use in diagnostics, as well as in research (e.g., sleep and circadian studies [2] or ergonomics [3]). Actigraph is typically a wrist-worn device, with a built-in accelerometer, battery and memory storage. Depending on an actigraph’s architecure, acceleration data can be digitized and stored in different modes, including zero-crossing mode (ZCM), time above threshold (TAT), digital integration (DI) [2,4] or raw acceleration [5].

As an actigraph can collect a longitudinal information about one’s movement profile, the application of actigraphy as a complementary or supplementary tool for diagnosis has been investigated for various sleep or circadian rhythm-related disorders. A recent review and meta-analysis by the American Academy of Sleep Medicine (AASM) Task Force [6] indicates the possible usefulness of actigraphy in, e.g., insomnia, circadian dysrhythmia or insufficient sleep syndrome.

For sleep-related actigraphic studies, one of the main objectives of the data analysis process is to distinguish between stages of sleep and wake during bedtime, with a general assumption of different movement intensity in each. Over the decades of research in the field of computational approaches for sleep-wake stages discrimination, various algorithms have been developed and investigated with respect to their accuracy, sensitivity or specificity in distinguishing the stages and bidirectional transitions between them. Part of such approaches relies mainly on an epoch-by-epoch movement intensity analysis, however, currently new methods based on machine learning techniques are being recognized as well [7,8,9].

In the work of Tilmanne et al. [10], several actigraphy sleep scoring algorithms were mentioned: Webster algorithm [11] as the earliest automatic approach; a group of Sadeh algorithms [12,13,14] and Cole-Kripke algorithm, which are the most recognizable; and Sazonov algorithm [15]. Another two algorithms, which seem to be less popular, yet can be found in research works, are: University of California San Diego (UCSD) algorithm [16] (e.g., in [17]) and Scripps Clinic [18] algorithms (e.g., in [19]). In a recent work of Palotti et al. [19], Authors compared the performance of some of the abovementioned sleep-wake scoring algorithms (including algorithms of: Webster, Cole-Kripke, Sadeh [13], Sazonov and Scripps Clinic) with more recent, machine learning-oriented approaches.

Instead of performance-oriented comparison, in our previous work [20] (in press) we performed a preliminary investigation of common mathematical features of two algorithms for actigraphic sleep-wake scoring—Sadeh [12] and Cole-Kripke [21]. In this article, the initial research is extended by four more algorithms: Scripps Clinic [18], Sazonov [15], UCSD [16] and Webster [11]. Additionally, we analyse their properties within the unified framework to show similarities and to unveil their common underlying major mode of operation—low-pass filtering.

## 2. Materials and Methods

### 2.1. Unified Framework Proposal

In this section, we present a unified view, linking together popular actigraphy algorithms in a mathematically coherent framework, composed of three steps:

1. Collapsing data into epochs.
2. Linear convolution with empirically chosen coefficients:
  (1) $$Y\left[n\right]=\sum_{i=-N}^{M}W\left(i\right)X\left[n-i\right]$$
  where W(i) is a vector of coefficients, *X* is the input signal, and N,M∈N.
3. Rescoring (optional), e.g. as in [21]: “After at least 4 min scored as wake, the next 1 min scored as sleep is rescored wake […]”.

Novelty of the proposed approach consists of:

1. Properly treating the first step as downsampling, which is a well-known signal processing procedure. This observation allows, e.g., to identify aliasing introduced by the procedures applied thus far, and design correct procedures.
2. Observing that the coefficients, used in different algorithms for the convolution step—W(i) in Equation (1)—in all cases actually result in a low-pass finite impulse response (FIR) filtering with very similar cutoff frequencies. This observation allows for an informed design of these filters for new algorithms using signal processing knowledge and tools, and an efficient analysis of the current approaches.

### 2.2. Defining Popular Actigraphy Algorithms under Unified Framework

In the following sections, we show how the discussed algorithms can be split into the three abovementioned steps, and how their defining equations are really a form of convolution of the signal and some empirically fitted kernel—effectively an FIR filter.

#### 2.2.1. Cole-Kripke Algorithm Family

Cole-Kripke [21], Webster [11], UCSD [16] and Scripps Clinic [18] algorithms start with signal downsampling, and then apply very similar equations on the resampled signal. This similarity allows to name these algorithms as Cole-Kripke family (descriptions and original equations are available in Appendix A.1 and Appendix A.2).

Cole-Kripke family equations are of identical form, differing in the number of elements in the sum and, obviously, values of the coefficients. In the following, we show that the similarities exceed significantly the mere mathematical form of the equations.

Step (2.) of the Cole-Kripke algorithm family can be defined as:

(2) $$D\left[n\right]=\sum_{i=-N}^{M}W\left(i\right)X\left[n-i\right]$$

where D[n] is the output value for epoch *n*, W(i) is a vector of coefficients, *X* represents the input signal and N,M∈N. In the following we show that algorithms from the Cole-Kripke family are equivalent to FIR filters.

A general FIR filter can be defined as:

(3) $$y\left[n\right]=\sum_{i=0}^{K}b\left(i\right)x\left[n-i\right]$$

where *y* is the output vector, *x* is the input vector, *b* is the vector of coefficients and *K* is the order of the filter. After changing indices in the sum in Equation (3), so that K−N=M, we obtain the following expression:

(4) $$y\left[n\right]=\sum_{i=-N}^{M}b\left(i+N\right)x\left[n-i-N\right]$$

After moving the whole output signal by *N* samples and changing the vector of coefficients so that W(i)≡b(i+N), we obtain the following equation, which is identical to step (2.) of the Cole-Kripke algorithm family (Equation (2)):

(5) $$D\left[n\right]=y\left[n+N\right]=\sum_{i=-N}^{M}W\left(i\right)x\left[n-i\right]$$

In other words, since moving the signal by few samples is irrelevant for further analysis, the actual action of step (2.) in the Cole-Kripke algorithm is equivalent to filtering the input signal with an FIR filter of order N+M. Rescoring rules (step (3.)) of a form “*after at least X minutes scored as A, the next Y minutes scored as A are rescored B*”, where *A* and *B* mean sleep or wake, may be applied to the filtered signal afterward.

#### 2.2.2. Sazonov Algorithm

Details of the Sazonov algorithm are described in Appendix A.4. Its output is given in terms of probability. At first glance, it appears to differ from the other discussed algorithms. Probability domain is [0,1] and Sazonov algorithm is basing on it, however, from logit function definition $\mathrm{logit}\left(p\left[n\right]\right)=\ln\frac{p[n]}{1-p[n]}\equiv \alpha \in \left[-\infty ,\infty \right]$. In this case threshold of p=0.5 is equivalent to α=0. This allows to easily eliminate the constant *d* from Equations (A6) and (A7) by adequately changing the threshold α, and also makes the similarity to Cole-Kripke algorithm more visible. Therefore, Equation (A7) will be referred to as follows:

(6) $$\alpha \left[n\right]=\sum_{i=0}^{8}A\left(i\right)\max\left[n-i\right]$$

where *A* is the vector of coefficients, max[n] is a maximum signal value in epoch *n*, α is the output of the algorithm. Equation (A6) should be understood in a similar fashion. This formulation includes steps (1.) and (2.) from Section 2.1, where max[n] implements “collapsing into epochs”, which are further convolved with A(i):

1. X[n]=max[n]
2. $\alpha \left[n\right]=\sum_{i=0}^{8}A\left(i\right)X\left[n-i\right]$

Now we see clearly, that the Sazonov algorithm is a member of the Cole-Kripke algorithm family—except for the application of rescoring rules, which are treated as optional either way. It is also worth noting, that Equation (A6), which was not used in the end, similarly can be perceived as a linear combination of three different Cole-Kripke algorithms with a different resampling method used within each of them.

#### 2.2.3. Sadeh Algorithm

At the beginning, it should be noted that both algorithms defined by Equations (A4) and (A5) contain an FIR component—for the first one it is an FIR filter of order 0, and for the second it is an FIR filter of order 10 combined with moving signal by 5 samples (similarly to the Cole-Kripke algorithm family). Mean from a few epochs can be represented as an FIR filter with all coefficients set to $\frac{1}{j+1}$, where *j* is the order of a filter. In both equations all components other than FIR filters are nonlinear. This allows to formulate the description of the Sadeh algorithm family as follows:

(7) $$PS\left[n\right]=\sum_{i=-N}^{M}W\left(i\right)X\left[n-i\right]+\sum_{i=0}^{K}C\left(i\right)\phi_{n}\left(i\right)+d$$

where the first component is equivalent to a FIR filter, while the second one is a linear combination of some nonlinear transformations $\phi_{n}\left(i\right)$. Finally, *d* is a constant and N,M,K∈N.

As explained further in Section 4, we could treat some of the nonlinear parts of the normalized Sadeh algorithm from Equation (7) as some sort of rescoring rules. However, there are at least two significant obstacles preventing that. Firstly, it is extremely nontrivial to convert input variables like sample variances into rescoring rules akin to Cole-Kripke rescoring rules. Secondly, rescoring rules are applied on the output of the filter post hoc, not during filtering. Additionally, these nonlinear components are obligatory—contrary to the rescoring rules. Although a part of the Sadeh algorithm is an FIR filter and it does fit our unified framework, additional nonlinear terms of Equation (7) prevent us from analyzing the Sadeh algorithm through the lens of the unified Equation (1), as it would be incomplete and could lead to false conclusions.

## 3. Results

### Properties of FIR Filters from the Cole-Kripke Algorithm Family

As presented in the previous section, all the discussed algorithms—except Sadeh’s—are equivalent to FIR filters. This simple observation allows for the first time to address the similarities and differences between these approaches in a unified and well-established framework. Furthermore, most importantly, the properties of FIR filters have been meticulously researched for decades, which greatly simplifies the physical interpretation of their properties.

**Figure 1:**
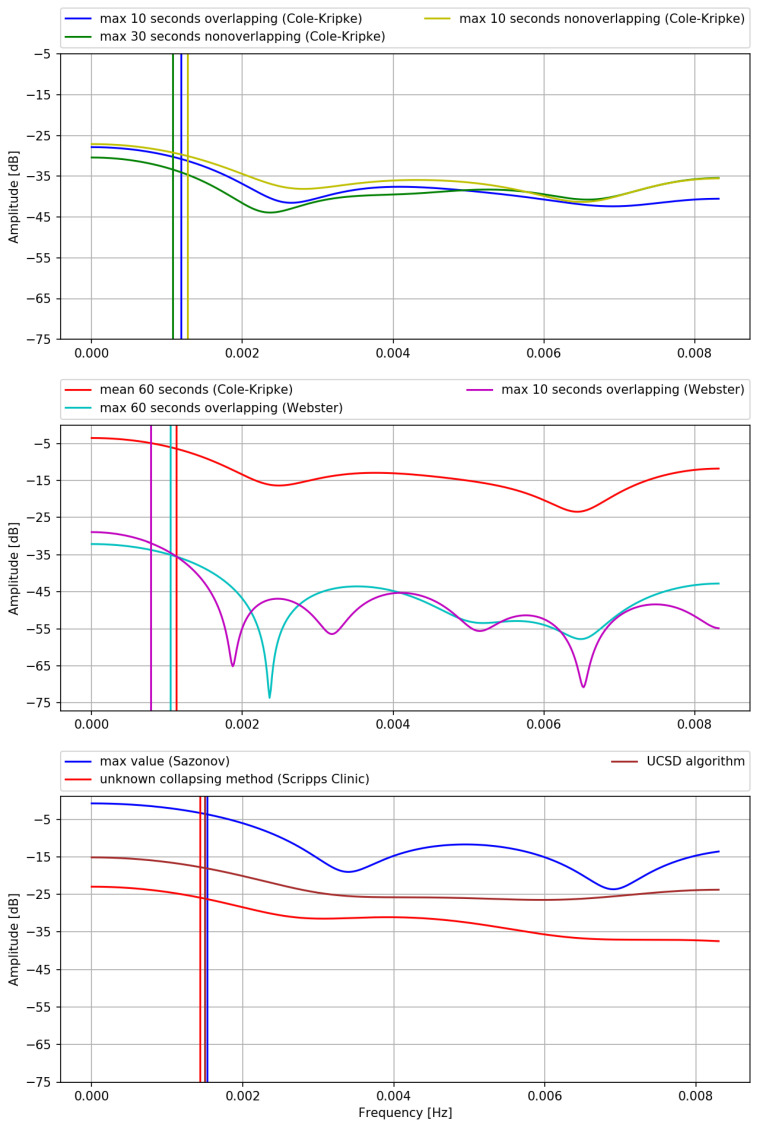
Transmittances of the FIR filters representing step (2.) of the Cole-Kripke algorithm family, aderived from the coefficients published by Authors. Vertical lines mark corresponding cutoff frequencies.

This fact is both illustrated and summarized in Figure 1, presenting the transmittances (frequency responses) of the FIR filters corresponding step (2.) of different (versions of the) algorithms. Original coefficients, as published by the authors of respective algorithms, are recalculated as corresponding FIR filter coefficients, according to the mathematical transformations of algorithms’ equations which were described in the previous sections. Transmittances of the filters are computed using the Scipy module (v. 1.7.1) in Python [22] in a following way: for a vector of frequencies *w* response $H(e^{iw})$ is returned, which in case of FIR filter of order *M* with coefficients W(i) means: $W\left(0\right)+W\left(1\right)e^{-iw}+\ldots +W\left(M\right)e^{-iwM}$. |H| is an estimate of the transmittance.

By inspecting Figure 1 and Table 1 we observe that all of these filters are low-pass filters with cutoff frequencies corresponding to a period of approximately 10–20 min. In the following, we logically split the filtering into two formal stages: weakening and the actual filtering. By weakening, we mean the decrease of the signal’s amplitude by a constant, introduced in the process of filtering by multiplying all the filter’s coefficients by the same value.

We discuss only one version of the UCSD algorithm (Appendix A.2) because the only difference between its versions is the value of multiplicative constant *P*, while all the $W_{i}$ coefficients are always the same (see Equation (A1)). This means that the only difference between versions of UCSD algorithm is the dB value at which the passband begins, while the shape of the transmittance or cutoff frequency are always exactly the same. In other words, all those versions of the UCSD algorithm are the same filters with different weakening.

The largest differences in transmittances, as observed in Figure 1, are between different algorithms (i.e., Webster vs. Cole-Kripke vs. Scripps Clinic vs. Sazonov vs. UCSD) as opposed to versions of the same algorithms (i.e., different resampling methods used in the same algorithm). This is most likely due to the use of different devices in each study, or, possibly, other different properties of signal, like sampling frequency before resampling.

However, the consequences of the presented result are even more severe, as it seems that not all statements made in [21] regarding limitations of their algorithms are true. An important, yet partially misleading statement made by Cole et al. [21] was that for each different method of collapsing data into epochs, new algorithms—i.e., new sets of coefficients—should be derived. This seems to be only partially true as inspecting Figure 1 one can see, that transmittances of filters derived for following three resampling methods: maximum 10 s of each minute considering overlapping epochs, maximum 10 s of each minute considering non-overlapping epochs and maximum 30 s of each minute considering non-overlapping epochs (see [21]) are virtually identical. Therefore, there is no need for using different filters for these different resampling methods, as opposed to Cole et al. statement. While there is a difference in the weakening stage of the filter with data resampled using mean vs. using max metrics, there is little difference between different methods of downsampling data using max metric with different epoch lengths, overlapping or not. Differences between mean and max metrics used during resampling data as described in the Appendix A.1 are due to the fact that the mean will be less (or equal, but this is rather not a case in real data) than the maximum value of a set. Since no matter what resampling method was used, data is normalized so that D[n]>1 means *wake*, weakening of the mean method should be lesser than of the max because the maximum value of a set is greater than or equal to the mean.

From the above we may infer that:

1. Discussed algorithms include a preprocessing stage, which consists of signal downsampling, but downsampling can have nontrivial sample selection. This procedure is not a standard resampling method and may introduce aliasing artifacts.
2. Sample selection metric (e.g., maximum, average) changes the overall signal weakening constant value, but the overall low-pass filtering cutoff frequency remains the same.
3. Weakening stage depends on certain hardware factors (like, e.g., sampling frequency).
4. Modern filter design tools could be used to design a low-pass filter instead of an empirical coefficient fit. Such a potentially unified algorithm design could be easily adaptable to different actigraphy hardware and sampling rates.

## 4. Discussion

All of the discussed algorithms rely on resampling input data as the first step of analysis, usually via some non-standard procedures. It is worth mentioning that these procedures (originally called “collapsing”) are potential sources of aliasing artifacts, as Nyquist theorem requires low-pass filtering before downsampling to avoid them.

All of the presented algorithms, except Sadeh’s (1989) [12] algorithm, can be interpreted as low-pass filters. Cutoff frequencies are very similar in all cases and correspond to periods from 10 to 25 min. Treating the central step of these algorithms as FIR filters should allow making better use of the signal processing theory, as there are well-established and optimized packages for filter usage and design. Furthermore, constructing similar algorithms in the style analogous to the Cole-Kripke algorithm family should be trivial and quick, while maintaining the assumption that the signal filtered with a low-pass filter should indicate *sleep* below some threshold and *wake* above it (or vice versa).

However, similarities are even broader than merely stemming from the presence of a low-pass FIR component. For all the discussed approaches it seems that the main goal of the procedures is to smoothen the input signal. Not only by low-pass filtering but also by other nonlinear operations such as the application of rescoring rules. Their smoothing action is for example due to the fact that short peaks of activity during sleep time will be rescored as sleep.

Sadeh algorithms can be in part described by the unified mathematical framework we proposed, although it can be done only for the linear part of the equations. Similar to the other discussed algorithms, Sadeh (1994) [13] contains a low-pass filter—in a form of a moving average.

**Figure 2:**
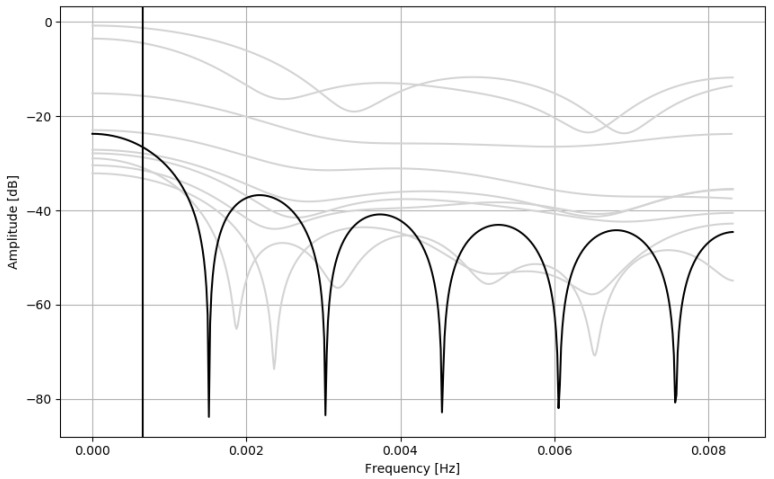
Transmittance of the FIR filter representing Sadeh 1994 linear terms of the equation (black), compared to the transmittances of the other algorithms discussed in this study (grey), the same as Figure 1. Cutoff frequency: 0.00065 Hz (period: 25 min 40 s).

Most nonlinear terms of Equation (A4) (Sadeh from 1989) include standard deviation of signal epochs. Standard deviation can be used to detect edges in a signal [23]. These terms are used to detect signal transients, and reduce their influence on the overall output (as mentioned in Appendix A.3), effectively smoothing the output signal. Sadeh from 1994 (Equation (A5)) uses a moving average, which is effectively a low quality low-pass FIR filter (Figure 2). Additionally, one of the nonlinear terms is also standard deviation of signal epochs, and some nontrivial epoch thresholding. All these considerations support the claim that Sadeh algorithms, like all other discussed approaches, aim to smoothen the actigraphic signal before applying some fixed threshold to discriminate sleep and wake.

Accuracies of the discussed algorithms, provided both by Authors [12,13,15,18,21] as well as some independent studies [10,24,25], are very close. It can be explained by the fact that the algorithms are in their core FIR filters with very similar transmittances. The unified view proposed in this paper allows to understand and analyze their properties within a coherent and well-established framework, in which not only the mathematical formulae seem to be just specific cases of one procedure, but also their intended modes of operation (i.e., resampling and smoothing) seem to be the same.

Finally, presented observations may possibly lead to an informed construction of algorithms for processing and analysis of actigraphic data, based upon a firm mathematical and signal processing background, with better argumentation and much less effort, as opposed to the purely empirical approach employed so far.

## Figures and Tables

**Table 1:** Cutoff frequencies (at −3dB) of low-pass filters corresponding to step (2.) of analyzed algorithms. Third column presents the corresponding periods in minutes.

Algorithm	Low-Pass Cutoff	Period
Cole-Kripke (max 10 s nonoverlapping)	0.00127 Hz	13 m 08 s
Cole-Kripke (max 10 s overlapping)	0.00119 Hz	14 m 02 s
Cole-Kripke (max 30 s nonoverlapping)	0.00107 Hz	15 m 31 s
Cole-Kripke (mean 60 s)	0.00112 Hz	14 m 50 s
Webster (max 60 s overlapping)	0.00104 Hz	16 m 00 s
Webster (max 10 s overlapping)	0.00078 Hz	21 m 20 s
UCSD	0.00150 Hz	11 m 09 s
Sazonov	0.00153 Hz	10 m 53 s
Scripps Clinic	0.00143 Hz	11 m 38 s
Average:	0.00121±0.00023 Hz	14 m 17 s ± 3 m 04 s

## Abbreviations

The following abbreviations are used in this manuscript:

FIR	Finite impulse response
PSG	Polysomnography
ZCM	Zero-crossing mode
TAT	Time above threshold
DI	Digital integration
AASM	American Academy of Sleep Medicine
UCSD	University of California San Diego

## Appendix A. Researched Algorithms

### Appendix A.1. Cole-Kripke Algorithm

Cole-Kripke algorithm, proposed by Cole et al. in 1992 [21], consists of three steps. The first step is performed via signal downsampling to $\frac{1}{60}$ Hz, described by original authors as “collapsing into 1 min epochs” of actigraphic signal gathered in the ZCM. The second step is defined by the following equation:

(A1) $$D\left[n\right]=P\sum_{i=-2}^{4}W_{i}X\left[n-i\right]$$

where D[n] is the output value of the second step of the algorithm, computed for given epoch *n*. Coefficients $W_{i}$ multiply values of the resampled signal from epoch X[n], and *P* is a normalizing multiplicative constant. The third step of the algorithm employs the rescoring rules to the vector obtained from Equation (A1).

The authors’ goal was to obtain an algorithm that would perform sleep-wake classification based on actigraphic data collected overnight. To provide the necessary variety of data for the determination of the coefficients $W_{i}$, groups of participants with mental or sleep disorders, as well as healthy participants, were selected to take part in an overnight recording of polysomnography (PSG) and actigraphy. Coefficients $W_{i}$ were obtained by fitting the actigraphic signal processed by this algorithm to the time series of 1-min epochs scored as *sleep* or *wake*, based on the PSG: D[n]>1 means *wake*, otherwise epoch is classified as *sleep*. With this constraint, the coefficients are fitted to maintain the highest agreement with the classification provided by the scored PSG recordings.

There are also two other assumptions about the input signal and the algorithm: sampling frequency of an actigraph, and the way of downsampling data (by taking mean or maximum value from an epoch, using different selections of subepochs in each epoch). Authors underline that after changing any of the above parameters, a new set of coefficients must be derived.

### Appendix A.2. Variations of the Cole-Kripke Algorithm: Webster, Scripps Clinic and UCSD Algorithms

The above-described Cole-Kripke algorithm shares a vast number of similarities with some other algorithms. Chronologically, the first algorithm of this kind, which we stumbled upon, is the Webster algorithm [11]. The main differences are the number of epochs taken into account in the equation defining the algorithm, and a different procedure of resampling data received from the actigraph.

Two variants of the Webster algorithm were proposed in [11]: the first was identical to Equation (A1), but with different coefficients (and different resampling method, as mentioned), and the second was expressed by the following equation:

(A2) $$D^{\prime }\left[n\right]=P^{\prime }\sum_{i=-4}^{5}W^{\prime }\left(i\right)X\left[n-i\right]$$

Another algorithm, almost identical to the previously described, is the Scripps Clinic algorithm [18]. This time the main difference lies in the fact that the input data was different—it was based upon the activity counts from a commercially available device of Actiwatch brand, and epochs are 30 s long. It is difficult to say how exactly these data are derived as it is not described in the article. Scripps Clinic algorithm is defined by the following equation:

(A3) $$D^{\prime \prime }\left[n\right]=P^{\prime \prime }\sum_{i=-2}^{10}W^{\prime \prime }\left(i\right)X\left[n-i\right]$$

Variables with primes in Equation (A2) or double primes in Equation (A3) have similar meaning as in Equation (A1). In both these algorithms, just like in the case of the Cole-Kripke algorithm, $D^{\prime }>1$ or $D^{\prime \prime }>1$ is interpreted as *wake*, otherwise *sleep*. Furthermore, in both cases some rescoring rules were used, of a form identical or similar to the one described in the Appendix A.1.

Yet another variation on Cole-Kripke algorithm is the UCSD algorithm [16]. In this case, the formula is identical to the Cole-Kripke algorithm (Appendix A.1), but the coefficients are different. Various sets of coefficients are proposed to enable the processing of different input data types. All of the signals, regardless of the data type, were gathered in 1-min epochs. Details about those coefficients are discussed in Section 3 of the article.

### Appendix A.3. Sadeh Algorithm

Sadeh et al. [12,13] proposed two algorithms, both being quite similar in assumptions. As the later algorithm [13] is of a more complicated formula, for now, the main focus will be on the earlier one [12] (The algorithm from 1994 [13] is more popular, and often referred to as “Sadeh algorithm”. We will refer to both of them as to “Sadeh algorithm” and differentiate them chronologically or through the reference to the original publication).

The version of the algorithm from 1989 [12] is defined by the following formula:

(A4) $$PS\left[n\right]=C_{1}+C_{2}X\left[n\right]+C_{3}S_{-5}+C_{4}S_{9}+C_{5}M_{2}+C_{6}S_{-2}$$

where PS[n] is the output value for given epoch *n*, $C_{i}$ are the coefficients, X[n] is the value of *n*-th epoch of the input signal, $S_{k}$ is the standard deviation of the following (if k>0) or preceding (if k<0) *k* epochs, and $M_{2}$ is the minimal value from the following two epochs. The algorithm returns *sleep* for *n*-th epoch if PS[n]>0, otherwise *wake*. Original coefficients proposed by authors are of following sign: $C_{1},C_{3},C_{4},C_{6}>0$ and $C_{2},C_{5}<0$. Therefore high standard deviation of signal is compensating other components and makes more likely given epoch to be clasified as sleep.

Similarly as for the Cole-Kripke algorithm, in the Sadeh algorithm coefficients, $C_{i}$ were derived by fitting data to a vector of binary classification (sleep/wake) obtained from single night PSG recordings parallel to the actigraphic recordings. However, in contrast to the Cole-Kripke algorithm, the variables (i.e., the operations computed on some number of epochs, as well as the number of epochs) used in the Sadeh algorithm are obtained by discriminant analysis, which from a given set of variables indicated those of the greatest relevance. Participants, in this case, included adults and children. Actigraph was gathering data in ZCM in epochs of 1 min length.

Later algorithm [13] is quite similar, and is defined by the following formula:

(A5) $$PS\left[n\right]=C_{1}^{\prime }+C_{2}^{\prime }{\mathrm{MEANW}}_{5}+C_{3}^{\prime }\mathrm{NAT}+C_{4}^{\prime }S_{-6}+C_{5}^{\prime }\mathrm{LOG}$$

where: MEANW${}_{5}$ is the mean value of the epoch *n*, 5 preceding and 5 following epochs, NAT is the number of epochs among the same 11 epochs taken for MEANW${}_{5}$, for which the value lies in the range [50,100], $S_{-6}$ is the standard deviation of the scored epoch and the 5 preceding, and LOG is the natural logarithm of the value in epoch n+1 (notation taken from [13]). Except for these differences, the algorithm is identical to the earlier Sadeh algorithm, that is the threshold and assumptions regarding the data remain the same.

### Appendix A.4. Sazonov Algorithm

In the following, the Sazonov algorithm, defined in [15], will refer to the explicit algorithm described below. Defining publication [15] reported also some applications of machine learning techniques, but this part will not be considered herein.

Sazonov algorithm was created for different devices than those described previously. The data used by Sazonov et al. [15] were gathered in the CHIME study [26,27], which investigated sleep in infants by means of PSG. Additionally, a simple 1-axis accelerometer was used, mounted on subjects’ bodies in standardized orientation. It was gathering raw acceleration data at a sampling frequency of 50 Hz. Data from this device was later discretized into an 8-bit signal, giving effectively a range of 256 unitless values, named “computer units”, and this discrete signal was used for the algorithm construction. Data were preprocessed by removing the DC constant and artifacts.

PSG data were scored in 30 s long epochs, so the data from the accelerometer were converted into 30 s long epochs too. It was done by using three metrics: maximum value of signal in given epoch *n* (max[n]), variance of signal in a given epoch (var[n]), and frequency of changes in a given epoch (freq[n]). The last one is, in fact, the same quantity as the previously mentioned ZCM, since it is defined as the number of zero crossings of the raw signal in a given epoch.

The Sazonov algorithm, in its first version, was defined by the following equation:

(A6) $$\mathrm{Logit}\left(p\left[n\right]\right)=\sum_{i=0}^{8}\left(A\left(i\right)\max\left[n-i\right]+B\left(i\right)\mathrm{var}\left[n-i\right]+C\left(i\right)\mathrm{freq}\left[n-i\right]\right)+d$$

where p[n] is the probability that, in the time corresponding to epoch *n*, the subject is sleeping, so p[n]>0.5 corresponds to sleep, and p[n]≤0.5 to wake. *A*, *B* and *C* are vectors of constants, and *d* is a scalar constant.

However, after post hoc analysis, the authors conclude that the variables var[n] and freq[n] are of relatively low significance, and reduce the algorithm to the following formula:

(A7) $$\mathrm{Logit}\left(p\left[n\right]\right)=\sum_{i=0}^{8}A\left(i\right)\max\left[n-i\right]+d$$

Just like the previously described algorithms, this one is also constructed by fitting the assumed formula (Equations (A6) and (A7)) to a vector consisting of sleep-wake classification derived from parallel PSG recording. In this case, logistic regression—as the formula of given equations suggests—was used to derive coefficients *A*, *B*, *C*, and *d*. This time authors do not explicitly give constraints, such as a statement that this algorithm is to be used only with a specific device, or specific sampling frequency. However, given that the device used in this study was not a standard actigraph, nor was its output standard, it may be assumed, that coefficients derived in [15] are also meant to be specific to this study or device.
